# Orthorexia nervosa, mindful eating, and perfectionism: an exploratory investigation

**DOI:** 10.1007/s40519-022-01440-4

**Published:** 2022-07-12

**Authors:** Molly Miley, Helen Egan, Deborah Wallis, Michail Mantzios

**Affiliations:** Department of Psychology, Birmingham City University, The Curzon Building, 4 Cardigan St., Birmingham, B4 7BD UK

**Keywords:** Orthorexia nervosa, Mindful eating, Perfectionism

## Abstract

**Purpose:**

Research has drawn associations between Mindful Eating (ME) and perfectionism in the aetiology and treatment of eating disorders (ED), but understanding into the relationship between these factors and Orthorexia nervosa (ON) is limited. The purpose of this research is to explore the relationship between perfectionism, ME, and ON.

**Method:**

Participants (*n* = 670) completed the Düsseldorf Orthorexia scale, the Mindful Eating Behavior scale, and the Big-Three Perfectionism scale Short-form, to reveal the relationship between ON, ME, and perfectionism. The relationship was assessed using correlational and regression analyses.

**Results:**

A positive association was observed between perfectionism and ON. Moreover, perfectionism demonstrated a significant negative correlation with three out of four ME facets, with “eating without distraction” displaying the highest correlation. The “eating with awareness” facet of ME demonstrated a significant relationship with ON, in a negative direction. An unexpected relationship was observed between the focused eating facet of ME and ON, with a positive association being found. A further regression analysis revealed both perfectionism and ME to predict orthorexic tendencies.

**Conclusion:**

These findings identify a relationship between ON, ME, and perfectionism. It offers suggestion for the complexity of ME, and how it should be recognised by its different components, estimating a differential predictability and estimation of ON. Further research is required to clarify the direction of causality in the relationships observed, to inform the clinical diagnoses and intervention of ON.

**Level of evidence:**

Level V, cross-sectional descriptive study.

## Introduction

The last decade has seen a rise in healthy decision-making, with particular emphasis on food-intake. Notably, the uptake of organic fruit and vegetables has seen a rapid increase [28], with people appearing to take greater responsibility for eating behaviours. These behaviours have been promoted through public health campaigns, including social media and school meal initiatives, encouraging the reduction of unhealthy foods from everyday diets [59, 68, 71]. While such programmes may facilitate healthy lifestyle changes, controlled, and restrictive eating behaviours have been implicated in the development of ON [62]. ON is a rather newly identified occurrence within eating behaviours [12], defined by preoccupation and obsessions with healthy eating [18, 58]. Such fixations with food can have detrimental consequences for physical health in terms of malnutrition, but can also negatively impact relationships, worklife, personal development, and general quality of life [10, 40]. For this reason, there is increasing support to justify ON classification within the ED spectrum [6] and the Diagnostic and Statistical Manual of Mental Disorders [DSM; 3]. As such, research must be undertaken to establish further evidence around its clinical significance and potential classification.

One area deemed influential upon disordered eating and dietary intake, is perfectionism [13, 23, 36, 42]. Being subjected and adhering to ‘perfect’ goals has become increasingly influential in predicting attitudes and health behaviours, with it being implicated in both mental illness and ED [17]. Perfectionism has been identified as a fundamental factor in the susceptibility of ON [25, 40]. Brown et al. (2012) reported a unique relationship between self-orientated perfectionism and possible ON symptomatology, namely, *preoccupation with eating* and *disordered eating behaviours*, independently. Moreover, Barnes and Caltabiano (2017) found that perfectionism contributed directly to ON; all dimensions of perfectionism positively correlated with ON and adherence to strict food rules. Valente et al. (2020) looked further into this relationship, utilising Instagram to obtain a sample of participants who had self-diagnosed themselves with ON, or utilised social media platforms to post about it. They observed a positive interaction between ON and perfectionism, with individuals explaining their orthorexic tendencies through perfectionist drives. Therefore, possessing perfectionist attitudes may predict self-reported ON. Perfectionism is often centred around a desire to control one’s environment and life events [72]. Therefore, having an orthorexic response to dietary intake may be an attempt to gain control over one’s own life and nutrition. The relationship between perfectionism and ON is, therefore, unsurprising, and may offer a potential area for future diagnosis classifications.

Regarding the relationship between perfectionism and disordered eating behaviours, mindfulness techniques are of increased interest. Mindfulness involves becoming aware and accepting of the present moment, whilst adopting a non-judgemental outlook on arising thoughts and feelings [37]. Such attitudes have been implicated in the reduction of both disordered eating and perfectionism [34, 77]. Olton-Webber, Hess and Ritchotte (2020) found mindfulness practices to reduce perfectionism after just 6 weeks. Moreover, James and Rimes (2018) found that not only did enhanced mindfulness decrease levels of clinical perfectionism, but also improved reported quality of life. This demonstrates the success of mindfulness techniques in the clinical demographic, offering support for similar techniques to be utilised in clinical disorders categorised by comparable perfectionistic attitudes, such as ON.

Mindfulness has been shown to negatively correlate with all dimensions of disordered eating [77], including dietary restriction [40] and obsessive preoccupations with food [19]. With both behaviours being experienced in those with ON [18, 58], it implicates the relevance of mindfulness in ON. Strahler [69] found mindfulness to moderate orthorexic eating, whilst also having protective abilities against ED pathology. Therefore, limited research implies the direct influence of mindfulness on ON, providing the foundations for further research into this area, with a more focused attempt on mindfulness that is specific to eating.

The practice of mindfulness techniques regarding eating behaviours is coined ME [44]. Through promoting the awareness and attentional facets of mindfulness towards the eating process, problematic eating behaviours can be addressed [75], although past definitions of ME have been challenged for being vague and unreliable [44]. Mantzios (2020) proposed that “*ME behaviour* is defined as the sustained attention on a sensory element of the eating experience (e.g. the taste), and a non-judgmental (or non-evaluative) awareness of thoughts and feelings that are incongruent to the sensory elements of the present eating experience.” (p. 3), which is more specific to the behaviour itself, rather than decision-making that is co-occurring when eating mindfully.

ME assists in the gradual change from external motivations to eat to internal motivations such as hunger when participating in ME interventions [46], promoting healthier eating behaviours [50, 52–54, 79], including an increased intake of fruit and vegetables [22, 26], and reductions in high sugar and energy dense food consumption [51, 56]. Research has also found a negative association between ME and motivations to eat palatable foods such as coping and social conformity [39, 45], fat and sugar consumption [48], and grazing [47, 49]. Pierson et al. [67] found ME to successfully reduce the intensity of food cravings and promote control over dietary intake, when used as an intervention. Egan and colleagues (2020) found further associations where ME related negatively to emotional eating [see also, 43], and other research highlighted the negative association to weight gain [55], and an impact of ME on reducing portion size and increasing self-regulation [33]; all evidence that could make it hard to distinguish between healthy eating behaviours and the promotion of ON. Although this knowledge may benefit specific problematic eating behaviours, such as those that contribute to obesity, a reverse effect may occur in the context of ON.

Indeed, ME has been implicated in the reduction of ED symptomatology and restrictive eating [1, 38]. Taylor, Daiss, and Krietsch (2015) found higher scores of ME to be associated with reduced ED symptomatology, specifically, preoccupation with food. This symptomatology is a core indication of ON [40], and so a similar interaction may be observed in this context directly. Kerin, Webb, and Zimmer-Gembeck (2018) reported the acceptance dimension of ME to predict a negative relationship with dietary restraint. Acceptance refers to being open and receptive to what one hears, thinks or sees, without attempting to alter it [37]. This acceptance allows individuals to acknowledge their body’s need for calories and food variety, which reaps positive effect on restrictive eating behaviours [1]. Therefore, acceptance may play an important role in the development of ON interventions, by addressing the restrictive eating components of ON [40]. Interventions should seek to promote acceptance of the body’s need for food variety, to prevent disordered eating behaviours.

With ON relating to a wide range of physical, social, and psychological disadvantages [40], its relevance within the clinical field is significant. However, as it is not yet recognised as an ED by the DSM-V, treatment and diagnosis remains largely misunderstood [63]. Therefore, the true prevalence of ON, assessed by applying commonly agreed on diagnostic criteria remains unclear. This highlights the demand for further research into ON, if understanding into aetiology and treatment is to progress. It has been suggested that both perfectionism and ME are relevant in the onset, maintenance, and treatment of disordered eating behaviours, although research has largely neglected the potential relevance to ON. Therefore, the present study utilised an exploratory design to investigate the relationship between ON, ME, and perfectionism.

## Method

### Participants

A total of 757 participants were recruited for the present study through volunteer sampling. Participants were invited to take part via social media platforms (Facebook, Instagram), and the survey was administered online via Qualtrics. The inclusion criteria ensured all participants were aged 18 or above, to secure fully informed consent and minimise distress to minors. Additional inclusion criterion ensured that participants did not have any history of eating disorder. This was to ensure that vulnerable and high-risk individuals were not subject to the sensitive questions regarding eating behaviours. This was also measured in the demographic questionnaire, where they were asked to indicate any ED diagnoses. Individuals that indicated a history of ED were removed from the final analysis, to ensure the results were not influenced by other disordered eating behaviours.

Due to the strict criteria implemented in the current study, 87 participants were excluded. This was a result of them having history of an ED (*N* = 7), being under the age of 18 (*N* = 2), or providing incomplete demographic data (*N* = 78). People without any entries on height or weight did not allow for BMI to be calculated, and the correct estimation as to where they would fall within any potential weight categories. Having an accurate estimate of the weight status of the population studied was of primary importance in reporting data that can be replicated in future studies. Data from the excluded participants were explored separately and demonstrated similar relationships. Therefore, data included in the study were obtained from a total of 588 female and 78 male participants (*N* = 670). The age of participants ranged from 18 to 74 (Mdn = 39 years, IQR = 29–51). Additionally, details of BMI were recorded (Mdn = 27.3, IQR = 23.8–31.9). G*Power and Cohen’s [16] guidelines were consulted and suggested that to achieve a small effect size, with alpha set at 0.01 and a power of 0.80, 698 participants were required to conduct a regression analysis with two independent variables. Over-recruitment accounted for an approximate 10% drop-out or missing data leading to exclusion from the final analyses. This ensured that even with missing data excluded, the sample size was sufficient for the purpose of the study.

### Materials

#### Demographics questionnaire

Participants were provided with a demographic questionnaire, gathering information on age, gender, height, weight, weekly exercise quantity, and smoking behaviours. Participants were also required to state whether they had ever received a diagnosis of ED, or mental health issue connected to body image/diet.

#### The Big-Three Perfectionism scale Short-form (BTPS-SF [24])

The BTPS-SF consists of 16 items, designed to assess self-reported levels of perfectionism. The items are arranged into three subscales: Rigid Perfectionism, Self-critical Perfectionism, and Narcissistic Perfectionism. Sample items include, ‘When I notice I have made a mistake, I feel ashamed’ and ‘It bothers me when people don’t notice how perfect I am’. Responses ranged from 1 (disagree strongly) to 5 (agree strongly), with a higher score indicating higher levels of perfectionism. With it displaying strong test–retest reliability and criterion validity [24], it is suitable for assessing levels of perfectionism in the target demographic. Within the present study, sample alpha was *α* = 0.899, *α* = 0.881 for Rigid Perfectionism, *α* = 0.890 for Self-critical Perfectionism, and *α* = 0.783 for Narcissistic perfectionism.

#### The Mindful Eating Behavior scale (MEBS [78])

The MEBS consists of 17 items designed to assess self-reported levels of ME. The items were arranged into four subscales: Focused Eating, Eating with Awareness, Eating in response to Hunger and Satiety Cues, and Eating without Distraction. Sample items include, ‘I notice how my food looks’ and ‘I multi-task when I am eating’. Responses ranged from 1 (never) to 5 (very often), with a higher score indicating a higher level of ME. This scale was selected due to its good internal consistency and convergent validity [78]. Within the present study, sample alpha was *α* = 0.831 for Focused Eating, *α* = 0.872 for Eating with Awareness, *α* = 0.855 for Eating in response to Hunger and Satiety cues, and *α* = 0.665 for Eating without Distraction.

#### The Düsseldorf Orthorexia scale (E-DOS [8])

The E-DOS consists of ten items designed to assess self-reported levels of orthorexic eating behaviours. The items form a single scale and include questions such as ‘I try to avoid getting invited over to friends for dinner if I know they do not pay attention to healthy nutrition’. Responses ranged from 1 (never) to 4 (always), with a higher score indicating stronger orthorexic tendencies. This scale possesses great concurrent reliability and construct validity [7, 11], with Cronbach’s *α* = 0.816 in the present study.

### Procedure

Participants responded to a range of online invitations via social media platforms. Interested individuals were presented with an information sheet and consent form online via the Qualtrics software. They then completed demographic questions, and were asked to formulate a unique identifying code, to maintain anonymity throughout. Upon completion, participants were presented with the three questionnaires, measuring perfectionism, ME, and self-reported ON tendencies. They were then directed to a debrief form. This provided participants with the researcher’s contact details and support services should they experience any distress.

## Results

### Sample characteristics

The sample consisted of 670 participants, with a median age of 39 years (IQR = 29–51). Female participants made up a large proportion of the sample (88%), and 20% of participants reported adhering to a weight loss diet. The median exercise engagement was 4 times per week (IQR = 2–12) and the median BMI was 27.3 (IQR = 23.8–31.9). Height and weight were self-reported. Finally, 84% of participants were White–British and 80% were non-smokers.

### Correlational analyses between ON, perfectionism, and ME

Correlational analyses were utilised to investigate the relationship between ON, perfectionism, ME, and BMI. It was found that the relationship between ON and perfectionism was significant, in a positive linear direction. However, despite showing a significant relationship, this association was weak to moderate, *r*(668) = 0.275, *p* < 0.001. Rigid perfectionism in particular showed the greatest correlation with ON *r*(668) = 0.296, *p* < 0.001. Moreover, perfectionism and ME were significantly associated. Analyses revealed significant negative correlations between the three dimensions of ME and perfectionism (See Table 1), with eating without distraction demonstrating the highest correlation *r*(668) = – 0.256, *p* < 0.001. The focused eating facet was not significantly related to perfectionism.

**Table 1:** Means, standard deviations, bivariate correlations between BMI, mindful eating, perfectionism, orthorexia nervosa, and each subscale

Questionnaire scores	1	2	3	4	5	6	7	8	9	10	*M*	SD
(1) BMI											28.19	5.51
(2) HS	– 0.302^b^										17.81	6.65
(3) FE	– 0.184^b^	0.367^b^									18.68	4.13
(4) EA	– 0.289^b^	0.296^b^	0.249^b^								11.82	2.94
(5) ED	– 0.181^b^	0.151^b^	0.178^b^	0.467^b^							20.84	4.17
(6) RP	– 0.031	– 0.018	0.108^b^	– 0.080^a^	– 0.132^b^						11.01	3.98
(7) SCP	0.085^a^	– 0.094^a^	0.023	– 0.236^b^	– 0.314^b^	0.631^b^					17.54	5.77
(8) NP	– 0.023	– 0.073	0.040	– 0.130^b^	– 0.128^b^	0.401^b^	0.373^b^				10.01	3.81
(9) E-DOS	– 0.026	– 0.044	0.122^b^	– 0.069^b^	– 0.070	0.296^b^	0.213^b^	0.163^b^			15.27	3.91
(10) BTPS	0.025	– 0.081^a^	0.065	– 0.198^b^	– 0.256^b^	0.831^b^	0.881^b^	0.687^b^	0.275^b^		38.57	11.01

*BMI* body mass index, *HS* Mindful Eating Hunger and Satiety subscale, *FE* Mindful Eating Focused Eating subscale, *EA* Mindful Eating Awareness subscale, *ED* Mindful Eating Distraction subscale, *RP* Rigid Perfectionism subscale, *SCP* Self-critical Perfectionism subscale, *NP* Narcissistic Perfectionism subscale, *E-DOS* English-Düsseldorf Orthorexia scale, *BTPS* Big-Three Perfectionism scale (Short-form)

^a^Is statistically significant at the 0.05 level

^b^Is statistically significant at the 0.01 level

Both the ‘hunger and satiety’ and ‘eating without distraction’ facets of ME were not significantly associated with ON in the present study. However, the ‘eating with awareness’ facet of ME demonstrated a weak but significant relationship *r*(668) = – 0.068, *p* < 0.001. The focused eating facet of ME showed a significant positive correlation with ON, *r*(668) = 0.122*, p* = 0.002, suggesting that the association between ME and ON is complex, and can be further understood by its different components (see Table 1).

All four facets of ME demonstrated a significant moderate negative relationship with BMI (see Table 1), implying that as ME increases, reported BMI scores decrease. This implicates ME in weight maintenance. The hunger and satiety element of ME showed the highest correlation *r*(668) = – 0.302, *p* < 0.001, demonstrating the differences in the relative contribution of the four facets of ME to BMI. Perfectionism and ON did not significantly relate to BMI.

### Regression analyses between perfectionism, ME, and ON

To determine the influence of all predictor variables to ON, a multiple regression analysis was conducted, using BMI and perfectionism as predictors.

The model obtained was statistically significant [F(2, 667) = 27.733, *p* < 0.001] and the predictive capacity calculated through *R*^2^adj was 0.077. This revealed both BMI and perfectionism to predict 7.7% of the variance in ON. Interestingly, results indicated that only perfectionism (*β* = 0.276, *p* < 0.001) significantly predicted ON, whereas BMI (*β* = – 0.033, *p* = 0.379) did not. Therefore, people experiencing higher levels of perfectionism may have an increased risk of ON, although BMI has no predictive abilities (see Table 2).

**Table 2:** Summary of multiple regression analyses for variables predicting orthorexia nervosa (*N* = 670)

Variable	*B*	SE *B*	Beta	*t*	Significance
BTPS	0.098	0.013	0.276	7.415	0.000
BMI	– 0.023	0.026	– 0.033	– 0.881	0.379

*BTPS* Big-Three Perfectionism scale (Short-form), *BMI* Body mass index

A further regression analysis was conducted with bootstrapping, involving each subscale of the predictor variables. The model obtained was statistically significant [*F* = (7, 662) = 11.342, *p* < 0.001], and the predicative capability calculated through *R*^2^adj was 0.098. This reveals that the subscales of both the Mindful Eating and Behaviour scale and the Big-Three Perfectionism scale predicted 9.8% of ON prevalence. Interestingly, results showed that rigid perfectionism (*β* = 0.250, *p* = 0.001) in particular, demonstrated a significant predictive relationship with ON, of a medium effect size. This was supported by the bootstrapped confidence intervals (CI). Moreover, the focused eating facet of ME, was also shown to significantly predict ON within the sample (*β* = 0.133, *p* < 0.001). This too was supported by the bootstrapped CI (see Table 3). The within-subjects design of this study indicates that as an individual traits of rigid perfectionism and focused eating behaviours increase independently, the more susceptible they are to ON. Specifically, one unit increase on the E-DOS, led to 0.250 SD increase in perfectionism, and 0.133 increase in focused eating, independently. This suggests that both areas are influential upon reported orthorexic eating behaviours.

**Table 3:** Summary of the criterion relationship with each predictor subscale

Variable	*B*	SE *B*	Standardised beta	*t*	Significance	BCa 95% confidence interval	
						Lower	Upper
FE	0.126	0.038	0.133	3.278	0.001	0.050	0.204
HS	– 0.047	0.028	– 0.067	– 1.657	0.098	– 0.107	0.013
EA	– 0.059	0.058	– 0.044	– 1.016	0.310	– 0.175	0.055
ED	– 0.019	0.040	– 0.021	– 0.481	0.631	– 0.102	0.065
RP	0.245	0.048	0.250	5.057	0.000	0.149	0.344
NP	0.040	0.042	0.039	0.963	0.336	– 0.052	0.134
SC	0.010	0.034	0.014	0.283	0.777	– 0.059	0.075

*FE* Mindful Eating subscale Focused Eating, *HS* Mindful Eating Hunger and Satiety subscale, *EA* Mindful Eating subscale Eating with Awareness, *ED* Mindful Eating subscale—Eating without Distraction, *RP* Perfectionism subscale, Rigid Perfectionism; *NP* Perfectionism subscale, Narcissistic Perfectionism; *SC* Perfectionism scale, Self-critical Perfectionism

## Discussion

The first aim of the present study sought to investigate the relationship between perfectionism and ON. As anticipated, a significant positive correlation was observed, suggesting that individuals experiencing the highly critical and judgemental beliefs associated with perfectionism are more susceptible to orthorexic eating behaviours. These findings were consistent for each dimension of perfectionism. This implicates the multidimensional concept of perfectionism in the onset and maintenance of problematic eating behaviours. This aligns with existing literature, which has repeatedly associated perfectionism with disordered eating [4, 13, 34] as well as ON [72]. Further regression analyses revealed rigid perfectionism to significantly predict orthorexic tendencies. This provides additional understanding into the observed interaction between perfectionism and ON. While each dimension of perfectionism is associated with ON, only the rigid perfectionism dimension predicts a significant increase in orthorexic eating behaviours. This suggests that perhaps the interaction between perfectionism and ON is complex, and should be understood by its varied dimensions.

The second aim of this study was to examine the relationship between ME and perfectionism. A negative correlation was anticipated, and predictions were mostly supported, with three out of four ME facets demonstrating a significant negative association with perfectionism. This suggests that being more mindful of eating behaviours is associated with reduced perfectionist attitudes. Such findings were consistent with previous literature into the broader concept of mindfulness, which has repeatedly demonstrated its negative association with perfectionism [34, 65]. With the present study extending the relevance of this interaction to the eating spectrum, this relationship offers clinical relevance and aligned support. Specifically, this knowledge may prove invaluable when evaluating ME interventions in a clinical context. It is already well established in existing literature that perfectionism is a recurrent risk-factor for eating disorders [66, 74], so the negative association between ME and perfectionism, suggests that perhaps encouraging ME in perfectionist individuals, may reduce disordered eating behaviours.

The observed relationship between ME and perfectionism can be understood through the differing perspectives on self-judgement. ME is defined by its non-judgemental focus [44], whereas perfectionism is defined by its highly judgemental and self-critical characteristics [27, 35]. It is, therefore, unsurprising that these concepts were negatively associated in the present study. An additional finding in the present study was the negative association between ME and observed BMI scores. Higher scores on ME were associated with a lower BMI. This is consistent with the notion that ME promotes healthier decision-making regarding eating behaviours and dietary selection [9], and supports the interaction observed in previous literature [21, 60]. With reduced over-eating relating to lower BMI scores [5], the results of the present study are unsurprising. This offers valuable insight for the clinical field in the treatment of problematic eating behaviours as identified by high BMI values. The healthcare system should promote all dimensions of ME to foster a healthy BMI, defined by the National Health Service as being between 18.5 and 24.9 [61], but similarly observe any interactions with ON. A BMI of 18.5 or below indicates being underweight, and displays potentially disordered eating behaviours [2, 61]. Those categorised as being ‘underweight’, are identified as higher risk for restrictive eating behaviours [2]. It is, therefore, important that healthcare professionals acknowledge that the process of making ‘healthier’ dietary decisions to achieve a reduced BMI may worsen orthorexic tendencies to restrict and consume only ‘healthy’ foods. This knowledge should be conveyed to those conducting potential ME interventions, to apply caution to its applicability, and closely monitor its progression. However, it is important to note that BMI is not always indicative of health, and fails to capture broader factors, including muscle mass and bone density [64, 76]. So, whilst BMI has a great utility in a clinical environment, it should not be relied upon as an indicator of health and wellbeing.

The final goal of this study was to investigate the interaction between ME and ON. Based upon research into other ED it was predicted that higher scores on ME would negatively correlate with orthorexic tendencies. Interestingly, it was only the ‘eating with awareness’ facet of ME, which is more of an indirect non-judgmental facet that supported these predictions. These findings suggest that promoting heightened awareness of eating during eating occurrences relates to reduced disordered eating behaviours. This is consistent with the success of mindfulness-based eating and awareness training [41]. ME focuses more specifically on the awareness of food sensations and satiety, as opposed to the types of food consumed [60]. This process is well-suited to those with ON, who tend to divert attention and awareness to foods nutritional quality. Through listening to hunger and satiety cues instead, it may potentially reduce restriction and promote healthier eating behaviours. Alternatively, ME also seeks to increase awareness of unmindful eating behaviours, and its subsequent consequences on health and wellbeing [43]. In the context of ON, perhaps increased awareness of the eating process and its consequences may lead to an informed reduction in unhealthy orthorexic tendencies, thus potentially promoting overall health and well-being. Healthcare workers should utilise this knowledge to design effective interventions for those presenting with orthorexic tendencies. Interventions should attempt to increase awareness towards the consequences of unmindful and orthorexic eating behaviours to reduce orthorexic symptomology.

An unexpected relationship was observed between the focused eating facet of ME, which is more of an attentive facet, and orthorexic tendencies. As focused eating increased, so did reported orthorexic tendencies, implicating focused eating in the maintenance of disordered eating behaviours. This positive association opposes literature into ME and its positive effect on disordered eating behaviours [70, 77], nevertheless, offers valuable insight into the complexity of ME. The observed relationship can be explained through the interaction between focused eating and memory recall [30].

Episodic memory has been identified as particularly influential among food-related decision-making, eating and satiety [15, 31, 32, 60], and literature suggests that memory can be improved through enhanced focus and attention [14], implicating ME in both memory recall and food-related decisions. Martin, Davidson and McCroy (2017) found that enhanced episodic memory was linked to significantly higher dietary control and restraint, implicating episodic memory in problematic eating behaviours, while other work has highlighted the implications for clinical populations such as anorexia nervosa patients [29]. Findings from Martin, Davidson, and McCroy (2017) support the results of the present study, explaining higher levels of orthorexic tendencies in terms of an increased focus on the eating process, and subsequent implications of episodic memory recall. This enhanced ability to recall food consumption is, therefore, associated with higher dietary control and restraint, both prominent symptoms of ON [40], but ON and episodic memory have not been explored in any scientific literature. Overall, both facets of ME associated to ON derived out of the behavioural aspects of the scale, and not the ‘decision-making’ facets, which should be considered when creating population relevant interventions. Interventions should attempt to decrease orthorexic behaviours through reducing focused attention to food, and decreasing memory recall as a result. Previous literature implies that this would reduce the restrictive and controlling behaviours associated with ON.

### Strengths and limitations

This study had several limitations that should be acknowledged. First, demographic characteristics may have influenced the observed results. With a large proportion of the sample identifying as female and White–British, caution should be applied when attempting to generalise these findings to the wider population. Future research should replicate these findings with a more diverse sample to aid a more universal understanding into the observed interaction. Finally, a small proportion of the sample are described as ‘elderly’ by the World Health Organisation (*n* = 27). These data, therefore, contains details on both adults and elderly participants, which was not considered in initial analyses. Research has shown that food intake and motivations to eat can decline with aging [20], suggesting that eating behaviours in this age category may be different to other age groups. This should be considered when interpreting the results.

There were also important limitations to discuss, independent of demographics. First, the online nature of data collection meant that BMI was self-reported. The accuracy of reported BMI’s can, therefore, not be validated, and caution should be applied when interpreting variable relationships with BMI. However, given the COVID-19 restrictions in place during data collection, it would have been unethical to obtain such data physically. Second, the cross-sectional nature of the study inhibits the ability to infer causality. Future research should build upon these findings, utilising an experimental design to allow greater exploration of the observed relationship. Third, despite the Düsseldorf Orthorexia scale being praised for its valid account for orthorexic tendencies, it is important to note that ON is not yet classified as a clinical disorder [63]. For this reason, there is currently no diagnostic criterion to aid in the diagnosis or identification of the disorder, and so measurement tools lack consistency. Scores of ON should, therefore, be interpreted with caution. Finally, despite achieving a relatively large sample size, the exclusion criteria disqualified 87 sets of data, leaving the sample size marginally below the 698-participant target. This should be acknowledged when interpreting the data, although, the sample size remains sufficient for the conclusions reached.

### What is already known on this subject?

Previous investigations indicate that mindfulness is associated with a range of problematic eating behaviours, including those that are restrictive in nature.

### What does this study add?

A significant association between ME and ON was observed, one that is unique to other research in the area. The focused eating dimension of ME was associated with increased orthorexic eating behaviours. These data confirm the complexity of ME as a concept, and offer further understanding into ON. To date, ON remains largely misunderstood in terms of diagnosis and treatment, a result of inconsistent and insensitive measurement strategies [73]. It is, therefore, hoped that this research will assist in establishing the clinical relevance and understanding of ON in association with and across different variables. Future research should build upon these findings, to assist in the clinical goal of understanding ON in terms of diagnosis and treatment.

## Data Availability

The datasets generated during and/or analysed during the current study are available from the corresponding author on reasonable request.

## References

[1] Albers S (2010). Using mindful eating to treat food restriction: a case study. Eat Disord.

[2] Alkazemi D, Zafar TA, Ebrahim M, Kubow S (2018). Distorted weight perception correlates with disordered eating attitudes in Kuwaiti college women. Int J Eat Disord.

[3] American Psychiatric Association (2013). Diagnostic and statistical manual of mental disorders DSM-5.

[4] Barnes MA, Caltabiano ML (2017). The interrelationship between orthorexia nervosa, perfectionism, body image and attachment style. Eat Weight Disord.

[5] Barry D, Clarke M, Petry NM (2009). Obesity and its relationship to addictions: is overeating a form of addictive behavior?. Am J Addict.

[6] Bartel SJ, Sherry SB, Farthing GR, Stewart SH (2020). Classification of orthorexia nervosa: further evidence for placement within the eating disorders spectrum. Eat Behav.

[7] Barthels F, Chard CA, Hilzendegen C, Stroebele-Benschoop N (2019). Psychometric evaluation of the English version of the Düsseldorf orthorexia scale (E-DOS) and the prevalence of orthorexia nervosa among a US student sample. Eat Weight Disord.

[8] Barthels F, Meyer F, Pietrowsky R (2015). Die Düsseldorf orthorexie skala-konstruktion und evaluation eines fragebogens zur erfassung ortho-rektischen ernährungsverhaltens. Zeitschrift für Klinische Psychologie Psychotherapie.

[9] Black DS, Sussman S, Johnson CA, Milam J (2012). Psychometric assessment of the mindful attention awareness scale (MAAS) among Chinese adolescents. Assessment.

[10] Bosi ATB, Çamur D, Güler Ç (2007). Prevalence of orthorexia nervosa in resident medical doctors in the faculty of medicine (Ankura, Turkey). Appetite.

[11] Brandl B, Greetfeld M, Hauner H, Holzapfel C, Hessler-Kaufmann JB, Meule A, Voderholzer U (2020). Measuring orthorexia nervosa: a comparison of four self-report questionnaires. Appetite.

[12] Bratman S (1997). Health food junkie. Yoga J.

[13] Brown AJ, Parman KM, Rudat DA, Craighead LW (2012). Disordered eating, perfectionism, and food rules. Eat Behav.

[14] Brown KW, Goodman RJ, Ryan RM, Anãlayo B (2016). Mindfulness enhances episodic memory performance: evidence from a multimethod investigation. PLoS ONE.

[15] Brunstrom JM, Burn JF, Sell NR, Collingwood JM, Rogers PJ, Wilkinson LL, Ferriday D (2012). Episodic memory and appetite regulation in humans. PLoS ONE.

[16] Cohen J (1992). A power primer: quantitative methods in psychology. Psychol Bull.

[17] Curran T, Hill AP (2019). Perfectionism is increasing over time: a meta-analysis of birth cohort differences from 1989 to 2016. Psychol Bull.

[18] Depa J, Barrada JM, Roncero M (2019). Are the motives for food choices different in orthorexia nervosa and healthy orthorexia?. Nutrients.

[19] Donini LM, Marsili D, Graziani MP, Imbriale M, Cannella C (2004). Orthorexia nervosa: a preliminary study with a proposal for diagnosis and an attempt to measure the dimension of the phenomenon. Eat Weight Disord.

[20] Donini LM, Savina C, Cannella C (2003). Eating habits and appetite control in the elderly: the anorexia of aging. Int Psychogeriatr.

[21] Durukan A, Gül A (2019). Mindful eating: Differences of generations and relationship of mindful eating with BMI. Int J Gastron Food Sci.

[22] Dutt S, Keyte R, Egan H, Hussain M, Mantzios M (2019). Healthy and unhealthy eating amongst stressed students: considering the influence of mindfulness on eating choices and consumption. Health Pyschol Rep.

[23] Egan SJ, Wade TD, Shafran R (2011). Perfectionism as a transdiagnostic process: a clinical review. Clin Psychol Rev.

[24] Feher A, Plouffe RA, Saklofske DH, Sherry SB, Smith MM, Wilson CA (2019). The big three perfectionism scale-short form (BTPS-SF): development of a brief self-report measure of multidimensional perfectionism. J Psychoeduc Assess.

[25] Fidan T, Ertekin V, Isikay S, Kirpinar I (2010). Prevalence of orthorexia among medical students in Erzurum, Turkey. Compr Psychiatry.

[26] Gilbert D, Waltz J (2010). Mindfulness and health behaviors. Mindfulness.

[27] Handorf A (2012). Mindfulness and perfectionism. Discussions.

[28] Harvard Medical School (2019). Bit by bit, Americans are eating healthier. Retrieved from https://www.health.harvard.edu/staying-healthy/bit-by-bit-americans-are-eating-healthier

[29] Hermans D, Pieters G, Eelen P (1998). Implicit and explicit memory for shape, body weight, and food-related words in patients with anorexia nervosa and nondieting controls. J Abnorm Psychol.

[30] Higgs S, Donohoe JE (2011). Focusing on food during lunch enhances lunch memory and decreases later snack intake. Appetite.

[31] Higgs S, Spetter MS (2018). Cognitive control of eating: the role of memory in appetite and weight gain. Curr Obes Rep.

[32] Higgs S, Williamson AC, Attwood AS (2008). Recall of recent lunch and its effect on subsequent snack intake. Physiol Behav.

[33] Hussain M, Egan H, Keyte R, Mantzios M (2021). Exploring the effects of mindfulness and self-distancing on chocolate intake after a negative state affect. J Cogn Enhanc.

[34] James K, Rimes KA (2018). Mindfulness-based cognitive therapy versus pure cognitive behavioural self-help for perfectionism: a pilot randomised study. Mindfulness.

[35] James K, Verplanken B, Rimes KA (2015). Self-criticism as a mediator in the relationship between unhealthy perfectionism and distress. Personal Individ Differ.

[36] Johnston J, Shu CY, Hoiles KJ, Clarke PJF, Watson HJ, Dunlop PD, Egan SJ (2018). Perfectionism is associated with higher eating disorder symptoms and lower remission in children and adolescents diagnosed with eating disorders. Eat Behav.

[37] Kabat-Zinn J, Hanh TN (2009) Full catastrophe living: using the wisdom of your body and mind to face stress, pain, and illness.Delta.

[38] Kerin JL, Webb HJ, Zimmer-Gembeck MJ (2018). Intuitive, mindful, emotional, external and regulatory eating behaviours and beliefs: an investigation of the core components. Appetite.

[39] Keyte R, Egan H, Mantzios M (2019). How does mindful eating without non-judgement, mindfulness and self-compassion relate to motivations to eat palatable foods in a student population?. Nutr Health.

[40] Koven NS, Abry AW (2015). The clinical basis of orthorexia nervosa: emerging perspectives. Neuropsychiatr Dis Treat.

[41] Kristeller JL, Epel E, Le A, Ngnoumen CT, Langer EJ (2014). Mindful eating and mindless eating: the science and the practice. The Wiley Blackwell handbook of mindfulness.

[42] Limburg K, Watson HJ, Hagger MS, Egan SJ (2017). The relationship between perfectionism and psychopathology: a meta-analysis. J Clin Psychol.

[43] Lofgren IE (2015). Mindful eating: an emerging approach for healthy weight management. Am J Lifestyle Med.

[44] Mantzios M (2020). (Re)defining mindful eating into mindful eating behaviour to advance scientific enquiry. Nutr Health.

[45] Mantzios M, Egan H (2018). An exploratory examination of mindfulness, self-compassion, and mindful eating in relation to motivations to eat palatable foods and BMI. Health Psychol Rep.

[46] Mantzios M, Egan H, Asif T (2019). A randomised experiment evaluating the mindful raisin practice as a method of reducing chocolate consumption during and after a mindless activity. J Cogn Enhanc.

[47] Mantzios M, Egan H, Bahia H, Hussain M, Keyte R (2018). How does grazing relate to body mass index, self-compassion, mindfulness and mindful eating in a student population?. Health Psychol Open.

[48] Mantzios M, Egan H, Hussain M, Keyte R, Bahia H (2018). Mindfulness, self-compassion, and mindful eating in relation to fat and sugar consumption: an exploratory investigation. Eat Weight Disord.

[49] Mantzios M, Egan H, Hussain M, Keyte R, Bahia H (2018). How does grazing relate to body mass index, self-compassion, mindfulness and mindful eating in a student population?. Health Psychol Open.

[50] Mantzios M, Giannou K (2014). Group vs. single mindfulness meditation: exploring avoidance, impulsivity, and weight management in two separate mindfulness meditation settings. Appl Psychol: Health Well-Being.

[51] Mantzios M, Skillett K, Egan H (2020). Examining the effects of two mindful eating exercises on chocolate consumption. Eur J Health Psychol.

[52] Mantzios M, Wilson JC (2014). Making concrete construals mindful: A novel approach for developing mindfulness and self-compassion to assist weight loss. Psychol Health.

[53] Mantzios M, Wilson JC (2015). Exploring mindfulness and mindfulness with self-compassion-centered interventions to assist weight loss: theoretical considerations and preliminary results of a randomized pilot study. Mindfulness.

[54] Mantzios M, Wilson JC (2015). Mindfulness, eating behaviours, and obesity: a review and reflection on current findings. Curr Obes Rep.

[55] Mantzios M, Wilson JC, Linnell M, Morris P (2014). The role of negative cognition, intolerance of uncertainty, mindfulness, and self-compassion in weight regulation among male army recruits. Mindfulness.

[56] Mason AE, Epel ES, Aschbacher K, Lustig RH, Acree M, Kristeller J, Daubenmier J (2016). Reduced reward-driven eating accounts for the impact of a mindfulness-based diet and exercise intervention on weightloss: data from the SHINE randomized controlled trial. Appetite.

[57] Martin AA, Davidson TL, McCrory MA (2017). Deficits in episodic memory are related to uncontrolled eating in a sample of healthy adults. Appetite.

[58] McComb SE, Mills JS (2019). Orthorexia nervosa: a review of psychosocial risk factors. Appetite.

[59] Mete R, Shield A, Murray K, Bacon R, Kellett J (2019). What is healthy eating? A qualitative exploration. Public Health Nutr.

[60] Moor KR, Scott AJ, McIntosh WD (2013). Mindful eating and its relationship to body mass index and physical activity among university students. Mindfulness.

[61] National Health Service (2019). What is the body mass index (BMI)? Retrieved from https://www.nhs.uk/common-health-questions/lifestyle/what-is-the-body-mass-index-bmi/

[62] Nazario B (2020). Orthorexia. Retrieved from https://www.webmd.com/mental-health/eating-disorders/what-is-orthorexia.

[63] Niedzielski A, Kaźmierczak-Wojtaś N (2021). Prevalence of orthorexia nervosa and its diagnostic tools—a literature review. Int J Environ Res Public Health.

[64] Nutall FQ (2015). Body mass index: obesity, BMI, and health: a critical review. Nutr Today.

[65] Olton-Webber S, Hess R, Ritchotte JA (2020). Reducing levels of perfectionism in gifted and talented youth through a mindfulness intervention. Gift Child Q.

[66] Penniment KJ, Egan SJ (2012). Perfectionism and learning experiences in dance class as risk factors for eating disorders in dancers. Eur Eat Disord Rev.

[67] Pierson S, Goto K, Giampaoli J, Hart S, Wylie A (2019). Impacts of a mindful eating intervention on healthy food-related behaviors and mindful eating practices among elementary school children. Calif J Health Promot.

[68] Rainey, S. (2016). How TV’s new queens of healthy eating are serving up hogwash: collagen soup. Eggs that are ‘astrologically harvested’ and why the Hemsley sisters don’t have a shred of expertise between them. Daily mail online. Retrieved from https://www.dailymail.co.uk/femail/article-3607736/How-TV-s-new-queens-healthy-eating-serving-hogwash-Collagen-soup-Eggs-astrologically-harvested-Hemsley-sisters-don-t-shred-expertise-them.html

[69] Strahler J (2020). Trait mindfulness differentiates the interest in healthy diet from orthorexia nervosa. Eat Weight Disord.

[70] Taylor M, Daiss S, Krietsch K (2015). Associations among self-compassion, mindful eating, eating disorder symptomatology, and body mass index in college students. Transl Issues Psychol Sci.

[71] Vaidyanathan, R. (2014). Can healthy eating be forced on children? BBC news. Retrieved from https://www.bbc.co.uk/news/world-us-canada-28355461

[72] Valente M, Brenner R, Cesuroglu T, Bunders-Aelen J, Syurina EV (2020). “And it snowballed from there”: the development of orthorexia nervosa from the perspective of people who self-diagnose. Appetite.

[73] Valente M, Syurina EV, Donini LM (2019). Shedding light upon various tools to assess orthorexia nervosa: a critical literature review with a systematic search. Eat Weight Disord.

[74] Wade TD, O’Shea A, Shafran R (2016). Perfectionism and eating disorders. Perfectionism, health, and well-being.

[75] Warren JM, Smith N, Ashwell M (2017). A structured literature review on the role of mindfulness, mindful eating and intuitive eating in changing eating behaviours: effectiveness and associated potential mechanisms. Nutr Res Rev.

[76] Weir CB, Jan A (2019). BMI classification percentile and cut off points.

[77] Wilson D, O’Connor EL (2017). Mindfulness, personality and disordered eating. Personal Individ Differ.

[78] Winkens LH, Van Strien T, Barrada JR, Brouwer IA, Penninx BW, Visser M (2018). The mindful eating behavior scale: development and psychometric properties in a sample of Dutch adults aged 55 years and older. J Acad Nutr Diet.

[79] Zervos K, Kolestski M, Mantzios M, Skopeliti N, Tsitsas G, Naska A (2021). An eight-week mindful eating program applied in a Mediterranean population with overweight or obesity: the EATT intervention study. Psychol Rep.

